# Waste not, want not: call to action for spinal manipulative therapy researchers

**DOI:** 10.1186/s12998-024-00539-y

**Published:** 2024-05-14

**Authors:** Sasha L Aspinall, Casper Nim, Jan Hartvigsen, Chad E Cook, Eva Skillgate, Steven Vogel, David Hohenschurz-Schmidt, Martin Underwood, Sidney M Rubinstein

**Affiliations:** 1 School of Allied Health, Murdoch University, Perth, Australia; 2 Medical Research Unit, Spine Centre of Southern Denmark, University Hospital of Southern Denmark, Middelfart, Denmark; 3 Department of Regional Health Research, University of Southern Denmark, Odense, Denmark; 4 Center for Muscle and Joint Health, Department of Sport Science and Clinical Biomechanics, University of Southern Denmark, Odense, Denmark; 5 Chiropractic Knowledge Hub, Odense, Denmark; 6 Department of Orthopaedics, Department of Population Health Sciences, Duke Clinical Research Institute, Duke University, Durham, NC USA; 7 Department of Health Promotion Science, Sophiahemmet University, Stockholm, Sweden; 8 Institute of Environmental Medicine, Karolinska Institutet, Stockholm, Sweden; 9 Naprapathögskolan, Scandinavian College of Naprapathic Manual Medicine, Stockholm, Sweden; 10 Research Centre, University College of Osteopathy, London, UK; 11 Pain Research, Department of Surgery & Cancer, Imperial College London, London, UK; 12 Warwick Clinical Trials Unit, Warwick Medical School, Coventry, UK; 13 University Hospitals of Coventry and Warwickshire, Coventry, UK; 14 Department of Health Sciences, Faculty of Science and Amsterdam Movement Science Research Institute, Vrije Universiteit, Amsterdam, the Netherlands

**Keywords:** Spinal manipulative therapy, Research waste, Methodology

## Abstract

**Background:**

Research waste is defined as research outcomes with no or minimal societal benefits. It is a widespread problem in the healthcare field. Four primary sources of research waste have been defined: (1) irrelevant or low priority research questions, (2) poor design or methodology, (3) lack of publication, and (4) biased or inadequate reporting. This commentary, which was developed by a multidisciplinary group of researchers with spinal manipulative therapy (SMT) research expertise, discusses waste in SMT research and provides suggestions to improve future research.

**Main text:**

This commentary examines common sources of waste in SMT research, focusing on design and methodological issues, by drawing on prior research and examples from clinical and mechanistic SMT studies. Clinical research is dominated by small studies and studies with a high risk of bias. This problem is compounded by systematic reviews that pool heterogenous data from varying populations, settings, and application of SMT. Research focusing on the mechanisms of SMT often fails to address the clinical relevance of mechanisms, relies on very short follow-up periods, and has inadequate control for contextual factors.

**Conclusions:**

This call to action is directed to researchers in the field of SMT. It is critical that the SMT research community act to improve the way research is designed, conducted, and disseminated. We present specific key action points and resources, which should enhance the quality and usefulness of future SMT research.

## Background

Globally in 2010, US$240 billion was spent on health research [1], most commonly on basic science and less on applied or clinical research. In 2009, Chalmers and Glasziou [2] estimated that 85% of funding for health research was wasted and defined four primary sources of research waste: (1) irrelevant or low priority research questions, (2) poor design or methodology, (3) lack of publication, and (4) biased or inadequate reporting. Disappointingly, they concluded in 2018 that while various initiatives had emerged to reduce waste and increase value, research waste remains a major problem [3]. Systematic reviews also contribute to this problem, with potentially few being both good quality and informative [4]. In recent years, the issue of research waste has been compounded by so-called ‘predatory’ journals with questionable or even absent peer-review practices, providing a platform for poorer quality research to be published under the guise of being adequately peer-reviewed [5, 6].

Since the issue of wasted healthcare research first received widespread attention in 1994 [7], initiatives to improve the quality and reporting of research have been introduced, such as the first CONSORT statement in 1996 for randomised controlled trials (RCTs) [8] and the first PRISMA statement in 2009 for systematic reviews [9]. Furthermore, since 2005 the International Committee of Medical Journal Editors (ICMJE) requires that clinical studies pre-register their protocol for two main purposes: (1) to limit publication bias, and (2) to prevent selective outcome reporting [10]. Most reputable journals, including those that commonly publish manual and physical therapy research [11, 12], state that they require clinical studies and systematic reviews to be pre-registered and to follow reporting guidelines such as the CONSORT and PRISMA Statements. However, enforcement of this remains unclear. For example, it appears common that articles lack a priori versions of trial protocols and statistical analysis plans even in high impact medical journals [13]. A 2020 review of reporting quality in manual therapy trials found that while reporting improved in some areas after implementation of the CONSORT Statement for non-pharmacologic interventions, it overall remained poor [14].

According to the World Health Organization (WHO), less than 3% of the grants funded by agencies reporting to the WHO in 2020 were awarded for research in the field of musculoskeletal disorders. Of those, only 36 (0.06% in total) addressed back pain [15] despite back pain being the number one cause of disease burden [16], defined as years lived with disability globally. A 2023 European Union (EU) report lists musculoskeletal pain as one of 12 main groups of proposed high-burden under-researched conditions, and states that the EU spends only 0.39% of health research funding on low back pain [17]. This highlights a critical issue for musculoskeletal researchers. While more resources should undoubtedly be allocated toward musculoskeletal research, we cannot afford to waste the limited resources we do receive!

Various interventions exist for the treatment of spinal pain, including spinal manipulative therapy (SMT). SMT is defined as manual therapy techniques that include high-velocity low-amplitude (HVLA) or thrust manipulation as well as low-velocity low-amplitude techniques such as mobilisation (or non-thrust manipulation) [18]. It is used by a wide range of health professionals, including chiropractors, physiotherapists/physical therapists, osteopaths, and naprapaths, for a variety of conditions, most frequently back and neck pain [18]. SMT is commonly recommended as a component of multi-modal care or second-line treatment for back and neck pain in international guidelines [19, 20]. However, the recommendations are typically based on low- or very low-certainty evidence, which appears to stem from a range of inadequacies in SMT research. Additionally, the underlying therapeutic mechanisms of SMT are inadequately understood [21, 22], and research is complicated by the multifactorial and poorly understood nature of spinal pain [23]. In short, there is a need for higher-quality research addressing clinical and mechanistic aspects of SMT [22, 24]; however, this research needs to be strategically focused so it addresses relevant research questions and identified knowledge gaps, and must be carried out and reported in such a manner that it truly contributes to the evidence base and not to the pile of research waste.

A multidisciplinary group of researchers with SMT and clinical experience have written this commentary to draw attention to the need for more high-quality research into SMT, primarily focusing on study design and methodology. We first discuss key issues in clinical then mechanistic SMT research. We then provide eight key action points and highlight various resources which we consider, if widely implemented, will reduce SMT research waste.

## Main text

### Clinical research

Trials investigating clinical effects of SMT are abundant, as are systematic reviews of these, especially for spinal pain. For example, a review protocol published in 2023 identified 85 systematic reviews which investigated the effects of SMT on patient-reported outcomes (including 442 trials) [25]. Despite the many papers, little progress has been made in identifying when SMT is likely to be beneficial and it remains unclear how SMT should be applied. The problem is compounded by systematic reviews offering conflicting conclusions often based upon evidence of low certainty, much of which can be ascribed to trials with a small sample size or high risk of bias.

In clinical SMT trials, there are often concerns with both internal and external validity. Regarding internal validity, reviews have identified frequent sources of potential bias. This includes inadequate randomisation and allocation concealment [26–31], which can introduce allocation bias and confounding through systematic differences in prognostic factors between groups. In addition, missing outcome data or loss to follow-up, especially if unbalanced between groups, is a common concern [26, 27, 29, 30]. Researchers should also be thoughtful about using appropriate outcome measures that are both valid and reliable. Potential bias in the reporting of results is also common [26, 27, 31, 32], where reporting may be influenced by knowledge of the results, such as omitting certain results or changing the primary outcome. Often these biases are difficult to assess due to poor reporting, resulting in higher risk of bias scores in reviews and, therefore, contributing to uncertainty. In particular, assessing reporting bias typically requires access to a detailed pre-registered statistical analysis plan, which is frequently absent.

Unclear or lack of blinding of participants, therapists, outcome assessors, and data analysers is also common [26–31]. This can lead to bias where knowledge of the intervention allocation leads to differences in participant or researcher behaviour (performance bias), or differences in how participants or researchers assess the outcome of interest (detection bias). We acknowledge that effective blinding of participants and therapists is particularly challenging for manual therapies [33]. Participant blinding is not always essential for effectiveness trials which assess real-world effects, while it is important for efficacy and mechanistic trials. In studies that do attempt participant blinding, few specifically assess and report on blinding success [32, 33], making it difficult to know whether expectations about outcomes were balanced between groups. Regardless, blinding of outcome assessors and data analysers is often very feasible and should be implemented.

As for external validity, descriptions of SMT tend to lack details (e.g., SMT target, intensity/dosage, and whether/how interventions were tailored to individuals) [34]. Such poor reporting makes it challenging for stakeholders to decide how applicable trial results are to their target setting and population. It also makes comparisons between studies and replication more difficult. Another issue affecting external validity is that procedures and interventions in SMT trials may poorly reflect how SMT is typically delivered in clinical practice. Researchers should carefully consider whether their research questions and design call for more experimental/controlled interventions or more pragmatic interventions.

Related to this is treatment fidelity, which is the degree to which an intervention was implemented and delivered in a study as intended [35]. Poor adherence to the intervention plan and poor performance of SMT affects both external and internal validity [36]. Data regarding fidelity are currently limited, especially for SMT, but fidelity is often not reported on in related fields [37] and health researchers appear to have poor knowledge or understanding of the concept [38]. In 2016, Karas and Plankis [39] made recommendations about how treatment fidelity can be implemented in manual therapy research. Methods to this end include standardising treatment dosages (e.g., time, repetitions, grade, force), as well as interactions between researchers and participants [39]. Strategies to enhance and monitor adherence can include the use of treatment manuals, specific therapist training, checklists, and observation of interventions [39]. As an emerging topic in ensuring methodological quality, future SMT researchers are encouraged to consider methods to enhance treatment fidelity and report on those explicitly.

A 2014 review reported a trend of improvement in the methodological quality of SMT studies, though trials remained small [40]. Despite this, authors of the 2019 updated Cochrane review on the effects of SMT for chronic low back pain [26] identified five new small pragmatic studies with a high risk of bias [41–45]. It was unclear in all five of these RCTs whether the treatment allocation was conducted properly, and only two had a registered protocol [41, 44]. Small studies may be underpowered, especially for secondary outcome measures, and this increases the risk of both type I and type II errors. There are also examples of small pilot studies [46] that make conclusions about SMT treatment effects. This may reflect misconceptions about the purpose of pilot studies [47] which are intended to assess the feasibility of conducting larger trials and should not be used to estimate treatment efficacy or effectiveness. These issues would suggest that we do not learn from past mistakes, or perhaps that messages about enhancing methodological quality have not been spread sufficiently.

While it is strongly encouraged that the results of all studies are published [2], poorer quality trials can confuse clinicians and patients and contribute to uncertainty in systematic reviews, rather than strengthening the evidence base. Poor quality trials also raise ethical concerns – it can be considered wasteful and inappropriate to expose participants to the risks and burden of participation when the results of that research might not be valuable or particularly informative, for example due to serious methodological flaws or particularly small sample sizes. To serve all the above needs, the solution is to improve the quality of trials being conducted in the first place, then to ensure they are published and reported appropriately.

Relatedly, it is important that clinicians and authors of case reports recognise that they can be valuable in specific circumstances, e.g., rare conditions or completely unexpected outcomes, but they are typically insufficient to inform treatment and policy decisions. Case reports should not be presented as evidence of SMT’s effect on particular outcomes.

Unfortunately, waste is not just limited to clinical trials but also occurs in systematic reviews and meta-analyses of SMT. One significant issue is the pooling of studies with highly heterogenous clinical populations (e.g., acute and chronic spine pain, radiculopathy, dysmenorrhea, and others [28]), different types of manual therapies [31], or different intervention dosage and frequency [31]. Pooling data for such varied conditions and interventions is problematic given the diverse aetiologies, treatment characteristics, and proposed mechanisms that may influence clinical outcomes. Furthermore, the type of meta-analysis should be appropriate; for example, the use of fixed-effects meta-analyses [31] may often be inappropriate in SMT research since it assumes minimal heterogeneity between studies [48]. Hence, issues around heterogeneity and appropriate statistical tests in reviews should be carefully considered to ensure reviews are informative for clinicians and other researchers. It is also important to recognise that the certainty of findings from a systematic review are directly influenced by the quality of the included studies, and SMT reviews frequently report low certainty due to the low quality of included studies [26, 27, 30].

### Mechanistic research

At present, the frameworks surrounding how SMT works are highly theoretical, highlighting our lack of understanding of SMT mechanisms. Many biomechanical and neurophysiological mechanisms have been investigated, including joint cavitations, joint forces, spinal stiffness, muscle activity, pain sensitivity, inflammatory markers, cortical activity, and autonomic activity. Reviews have highlighted the many knowledge gaps and weaknesses in the literature [21, 22, 49–51]. These include short-term outcomes, a lack of translational research and demonstrated clinical relevance, and inadequate control for contextual factors.

The typical translational workflow of research, advancing from basic science with animal or laboratory models to humans, then clinical populations and finally real-world settings [52], is often not followed in mechanistic SMT research. For example, many human studies assessing pain inhibition using quantitative sensory tests have been summarised in multiple systematic reviews [53–57], yet few animal studies directly assess painful stimuli after joint manipulation and attempt to illustrate related neurophysiological pathways [21, 50, 58]. There is only one study in a pragmatic setting involving more typical clinical care [59]. Therefore, we can observe differences following SMT but, since there are few studies in the early and later stages of the translational research workflow, we do not know exactly what physiological mechanism/s drive this change and whether it is related specifically to SMT.

Relatedly, research often fails to bridge the gap between mechanisms and their clinical relevance [21, 22, 49, 51]. This is an important step in determining the significance of an observed biomechanical or neurophysiological change, and therefore the value of continuing to research it. This can be explored in various ways, including testing mechanisms in clinical populations and clinical settings, and correlating biomechanical or neurophysiological outcomes with clinical outcomes. Returning to the example of quantitative sensory testing research, several recent studies have found that changes in pressure pain detection threshold after SMT may not correlate with patients’ pain intensity or self-reported improvement [59–61], suggesting that this particular outcome may have limited clinical relevance in our pursuit of understanding specific effects of SMT.

Mediation, moderation, and time-lagged analyses all offer more advanced approaches to studying the clinical relevance of mechanisms, allowing causal inferences to be made. These methods have a long history of use in psychology [62–64], but few studies have used them to elucidate SMT mechanisms [65]. A mediator is a variable measured during treatment that affects (or mediates) the response to treatment [66]. In contrast, a moderator variable is a baseline characteristic that affects response to treatment [66]. These are both distinct from predictors, which are baseline variables that predict outcome independent of any treatment effect. Time-lagged analyses use longitudinal data to explore how the relationships between variables may change over time [67]. Simplistically, these types of analysis could help identify mechanisms that influence clinical outcomes, or that precede and predict clinical outcomes.

Mechanistic studies are often focused on extremely short time frames compared to clinical trials; a common design is to measure an outcome before and almost immediately after a single-session intervention. For example, studies have observed immediate changes in various serum biomarkers [68, 69] and neurological activity [70, 71]. Numerous reviews have called for longer-term outcome assessment in mechanistic SMT research [49, 72–74]. Short-term outcome studies can be informative, especially to look for early indications of change, and are relatively easy to conduct (important for under-resourced researchers). However, they can also severely limit the usefulness of findings given that changes may be highly transient and their relevance to clinical outcomes cannot be assumed. If the mechanism of interest appears to be relevant, such short-term studies should be complemented with more pragmatic studies that investigate longer time frames and clinical relevance.

Many mechanistic SMT studies on humans do not adequately account for contextual factors, including participant expectations [21, 49]. For example, some studies use control interventions of passive joint movement [75, 76] or touch without attempting to blind participants [77] or are conducted on likely non-naïve healthcare students [78–80]. Control interventions in efficacy or mechanistic trials (also known as ‘sham’ controls) that do not attempt to mimic SMT, such as detuned ultrasound, have also been criticised [33]. Such approaches likely result in unequal participant expectations and differences in contextual factors. Given that context-dependent effects can produce wide-ranging neurophysiological changes and clinical effects [81–84], this makes it difficult to attribute observed changes as specific effects of SMT. While there are inherent challenges to this in non-pharmacological interventions, high-quality control interventions to balance expectancy effects and attempts to blind participants are important if we are to understand whether mechanistic (or clinical) responses to SMT are related to the intervention itself or to contextual factors surrounding the delivery of SMT [85]. However, such control interventions are not always feasible or appropriate depending on the trial design and research questions.

### Where to next

Given the limited resources and funding opportunities available for SMT research globally and across professions, it is critical that the SMT research community act to enhance the way our research is designed, conducted, and disseminated. This stands to benefit the principal stakeholders in SMT research: clinicians and patients. Adhering to best practice principles for research conduct and reporting is critical, and there are an increasing number of resources specific to studies of musculoskeletal pain, manual therapies, and SMT to support this. Development of methodological guidelines or tools specific to SMT could also advance this field.

There is also a need to collaborate. Working with researchers with diverse skills and from various disciplines and institutions provides wide-ranging benefits, such as improved possibilities to acquire funding, sharing of expertise and resources, more innovative and flexible thinking, and higher impact [86–88]. Collaboration can also mean involving clinicians and/or patients in the design and conduct of studies, which helps ensure research questions and methodologies are relevant and impactful to stakeholders [89, 90].

Nonetheless, we acknowledge there are barriers to achieving this goal. Perhaps most important is the limited funding available for musculoskeletal research. For SMT specifically, many studies are conducted with no or minimal funding, or as research student projects. This hampers efforts to conduct large and robust trials, limiting access to resources and methodological expertise. Building effective inter-disciplinary collaborations and teams that can be competitive in seeking funding is a critical step in addressing this issue. Supporting future SMT researchers is also important, and at times this can lead to conflict between the needs of the novice or early career researcher and the desire to conduct highly powered and impactful research. A key aspect of early career SMT researcher experiences should focus on the development of knowledge, skills, and values required to conduct high-quality research. This includes instilling a deep commitment to academic rigour, integrity, and transparency. For research students wishing to develop skills in SMT trials, it is important that adequate resources and supports are available to ensure the trials are conducted rigorously. This might mean seeking out well-funded teams, especially for clinical research. Crucially, future SMT researchers need the training and support to obtain competitively awarded research funding.

**Fig. 1:**
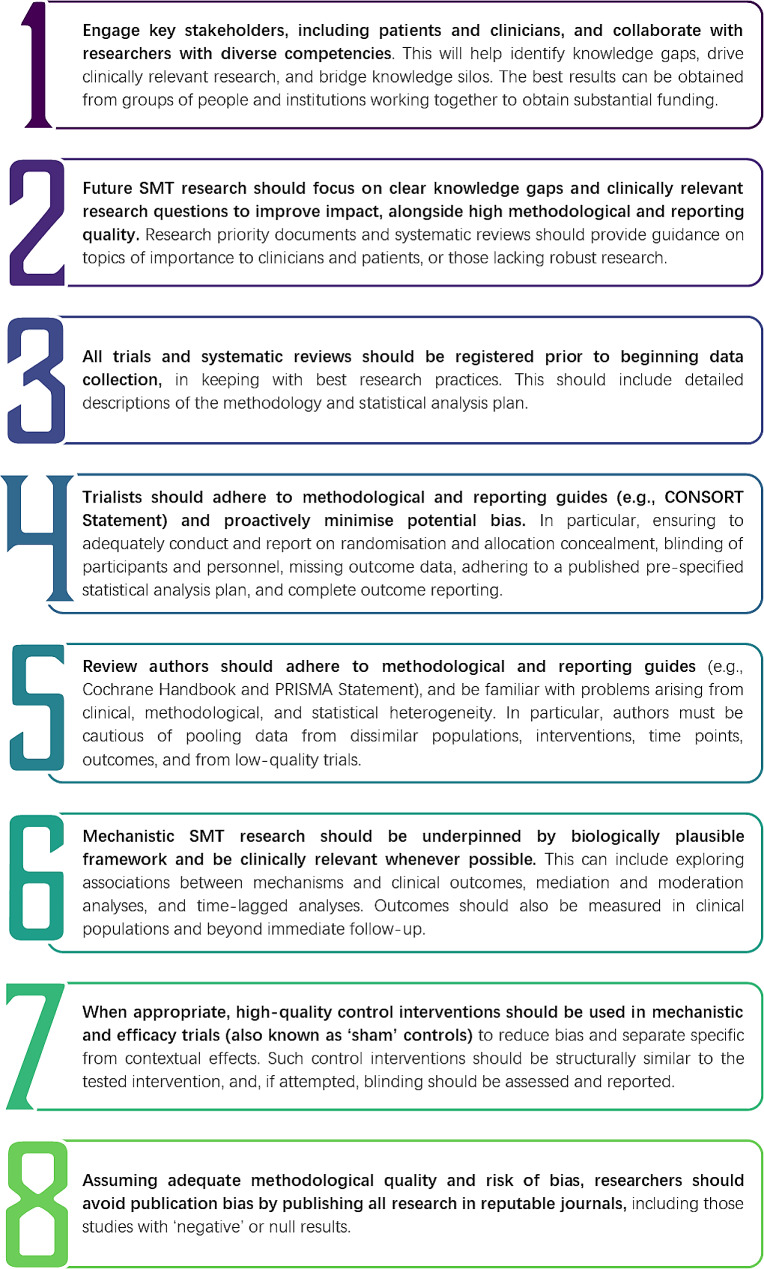
Key action points for spinal manipulative therapy researchers. Abbreviations: SMT = spinal manipulative therapy

Despite the barriers, the problem of waste in SMT research is largely avoidable. We call on SMT researchers, supervisors, and funding bodies across all professions to prioritise high-quality research through appropriate research questions, design and methodology, publication, and reporting. In Fig. 1 we summarise eight key action points arising from this paper. We also highlight resources intended to improve the quality of research in Table 1.

**Table 1:** Important resources for spinal manipulative therapy researchers

**EQUATOR Network** [96]	The EQUATOR Network provides robust **reporting guidelines** for many study types to improve transparency and quality of reporting in health research. While not methodological guidelines, these may assist when developing study protocols. Includes: • **CONSORT 2010 Statement** [97] and its extensions designed for randomised trials and sub-types, • **TIDieR checklist and CoPPS Statement** which deal specifically with reporting of interventions and controls, • **CIRCLe SMT guideline** [98] for detailed reporting of SMT interventions, • **STROBE Checklist** for observational epidemiology studies, • **PRISMA 2020 Statement** [99] for systematic reviews, and • **Statements for other study types including qualitative studies and case reports.**
**Study Registries**	Various study registries exist for the primary purpose of transparent pre-registration of study aims and methods. These can be used to provide evidence of pre-registration to journals, assess aspects of risk of bias, and encourage reporting of results. Includes: • **ClinicalTrials.gov** and **ANZCTR** for registration of clinical trials (interventional and observational), • **PROSPERO** for registration of health-related systematic reviews, • **Open Science Framework (OSF)** for registration of any study types, and • The World Health Organization maintains the International Clinical Trials Registry Platform (ICTRP).
**Risk of Bias**	Cochrane have developed comprehensive risk-of-bias tools which are designed for assessing risk of bias of studies included in systematic reviews. These tools can also be used when designing studies to identify methodological choices that reduce potential bias. • **RoB 2** [100] for randomised trials and sub-types, and • **ROBINS-I** [101] for non-randomised studies of interventions.
**Other Resources**	• **Fostering patient involvement**: Arumugam et al. [89] provide a practical resource for involving patients and the public in research. • **Conducting systematic reviews and meta-analyses**: the Cochrane Handbook for Systematic Reviews of Interventions [102] provides extensive methodological guidelines. • **Designing pragmatic trials**: a dedicated IMMPACT statement [103] summarises relevant methodological considerations for the design and conduct of pragmatic trials of pain interventions. • **Enhancing control interventions**: the CoPPS statement [85] makes “recommendations for designing, conducting, and reporting control interventions” that are relevant for SMT research. • **Monitoring treatment fidelity**: Karas et al. [39] make recommendations for implementing and reporting on treatment fidelity for manual therapies.

Abbreviations: SMT = spinal manipulative therapy.

In support of this Call to Action, the authors of this commentary are planning to develop a series of papers addressing specific issues relating to waste in SMT research. While numerous prior papers have tackled this issue in the biomedical field generally [91–95], future papers will take a focused view on specific problems and practical solutions in our field of SMT research.

## Conclusion

This call to action is directed to researchers in the field of SMT. It is critical that the SMT research community act to improve the way research is designed, conducted, and disseminated in order to enhance the usefulness of SMT research for clinicians and patients. In pursuit of this goal, we present eight key action points and various resources that are relevant for SMT research.

## Data Availability

Not applicable.

## Abbreviations

HVLA: High-velocity low-amplitude
RCT: Randomised controlled trial
SMT: Spinal manipulative therapy
